# Surfactant Protein D Is Altered in Experimental Malaria-Associated Acute Lung Injury/Acute Respiratory Distress Syndrome

**DOI:** 10.1155/2019/9281605

**Published:** 2019-08-01

**Authors:** Chuchard Punsawad, Parnpen Viriyavejakul, Tachpon Techarang

**Affiliations:** ^1^School of Medicine, Walailak University, Nakhon Si Thammarat, 80160, Thailand; ^2^Tropical Medicine Research Unit, Research Institute for Health Sciences, Walailak University, Nakhon Si Thammarat, 80160, Thailand; ^3^Department of Tropical Pathology, Faculty of Tropical Medicine, Mahidol University, Bangkok, 10400, Thailand

## Abstract

Surfactant protein D (SP-D) is in the collectin family of C-type lectins and plays an important role in the regulation of inflammation and the innate immune defense against pathogens. This protein has been proposed as a biomarker for acute lung injury. However, the expression of SP-D in the lung and the circulating levels of SP-D during malaria infection have received limited attention. Therefore, the aim of this study was to determine the location and expression of the SP-D protein in lung tissue and to measure the plasma level of SP-D in experimental malaria-associated acute lung injury/acute respiratory distress syndrome (ALI/ARDS). Malaria-infected mice induced by* Plasmodium berghei *ANKA were classified into two groups, namely, the ALI/ARDS and non-ALI/ARDS groups, according to lung histopathology. The lungs of uninfected mice were used as a control group. The location and expression of SP-D in the lung tissues were investigated by immunohistochemical staining and Western blot analysis. In addition, the level of SP-D in plasma and lung homogenate was measured by an enzyme-linked immunosorbent assay. Immunohistochemical staining of SP-D was significantly increased in the lung tissues of the malaria-infected mice in the ALI/ARDS group compared with that in the malaria-infected mice in the non-ALI/ARDS group and the mice in the control group (*p* < 0.05). The levels of SP-D in the plasma and lung homogenate were significantly increased in the malaria-infected mice in the ALI/ARDS group compared with those in the malaria-infected mice in the non-ALI/ARDS group and the mice in the control group (*p* < 0.05). There was a significant positive correlation between SP-D in the plasma and SP-D in the lung homogenate (*r*_*s*_ = 0.900,* p* = 0.037). In conclusion, this study demonstrated increased expression levels of SP-D in the lung tissue and high levels of plasma SP-D in the malaria-infected mice with ALI/ARDS compared with those in the mice in the other groups. The current study supports that the elevation of the plasma SP-D level may provide useful biological confirmation of the diagnosis of ALI/ARDS during malaria infection.

## 1. Introduction

Malaria-associated acute lung injury/malaria-associated acute respiratory distress syndrome (ALI/ARDS) is prevalent in malaria-endemic areas, with a mortality rate of approximately 80% [1]. Previous studies reported that the mechanisms of inflammation and increased endothelial permeability contribute to the pathogenesis of ALI/ARDS in human and animal models with malaria infection [1–3]. To date, many researchers have attempted to discover potential biomarkers for evaluating and monitoring pulmonary complications, especially ALI/ARDS, during malaria infection. A variety of biomarkers have been used to detect and monitor acute lung injuries in experimental animal models and humans [4, 5]. Several studies have suggested that the surfactant protein D (SP-D) level is a useful biomarker of lung injuries in rodents [6–11] and patients with ALI [5, 12–14].

SP-D is a collagenous calcium-dependent lectin that is involved in surfactant homeostasis and the innate immune defense within the lung [15–19]. This protein binds to surface glycoconjugates expressed by a wide variety of microorganisms and to oligosaccharides associated with the surface of various complex organic antigens [15, 17]. In lung tissues, SP-D is constitutively synthesized and secreted by alveolar type II cells, bronchiolar epithelial cells that are known as Club cells, and alveolar macrophages [16, 20, 21], but SP-D is also expressed in the epithelial lining of various organ systems, including circulatory, integumentary, digestive, and genitourinary tracts, in humans [15, 19, 21]. In humans, SP-D is elevated in the serum and plasma of patients with ALI/ARDS [12–14, 22]. In a murine model of ALI, increased levels of SP-D have been observed in the serum and bronchoalveolar lavage fluid (BALF) after treatment with bleomycin [9, 10] or lipopolysaccharide [8], suggesting that SP-D can be used as a biomarker for monitoring the development of ALI. In terms of malaria infection, the level of serum SP-D in mice is correlated with ALI in a severe malaria C57BL/6 mouse model [6]. However, little information on the expression of SP-D in the lungs and SP-D levels in plasma of patients with ALI/ARDS during malaria infection has been reported. Nevertheless, it is hardly possible to obtain both lung sections and plasma from human patients simultaneously. Therefore, this study aimed to determine the location and expression of SP-D in the lung tissues of mice with malaria-associated ALI/ARDS by immunohistochemistry staining and Western blot analysis and to measure the level of SP-D in the lung tissues and plasma by an enzyme-linked immunosorbent assay (ELISA).

## 2. Materials and Methods

### 2.1. Animal Model of Malaria-Associated ALI/ARDS

All animal procedures and protocols were approved by the Animal Ethics Committee from Walailak University (protocol no. 003/2018). All experiments were performed in accordance with relevant guidelines and regulations. Six- to ten-week-old male DBA/2 mice were purchased from BioLASCO Co., Ltd. (Taipei, Taiwan). The mice were maintained in the Animal Experiment Building at Walailak University and housed in a conventional animal house with free access to food and fresh water. The animal house was set at 22 to 24°C with a standard 12 hr light/dark cycle. The mice were intraperitoneally injected with 1 × 10^6^* Plasmodium berghei* ANKA-infected red blood cells to induce ALI/ARDS according to methods described in previous studies [23, 24]. The malaria parasite was obtained through BEI Resources, NIAID, NIH:* P. berghei*, Strain ANKA, MRA-311, contributed by Thomas F. McCutchan. The control mice received saline solution via intraperitoneal injection. After infection, parasitemia was determined daily by microscopic analysis of thin blood smears from tail blood after Giemsa staining. All the infected mice with signs of ALI/ARDS and the control mice were sacrificed at day 13 after infection. Blood was collected by cardiac puncture, and plasma was prepared and stored at -20°C until it was used for SP-D measurements. The lung tissues were removed immediately for immunohistochemistry and Western blot analyses. The lung tissues of the malaria-infected mice were categorized into two groups (ALI/ARDS and non-ALI/ARDS groups) in accordance with the evaluation of lung histopathology by an expert pathologist. Specifically, the lungs with ALI/ARDS were diagnosed based on the observation of pulmonary edema, alveolar hemorrhage, alveolar septal thickness, and hyaline membrane formation. Only 50% (n = 5 mice out of 10) of the* P. berghei*-infected mice developed ALI/ARDS.

### 2.2. Immunohistochemistry Staining (IHC)

Sections of formalin-fixed and paraffin-embedded lung tissues were incubated at 60°C for 1 hr followed by deparaffinization and rehydration. After rehydration, endogenous peroxidase activity was blocked with 3% hydrogen peroxide for 10 min. Subsequently, the lung sections were incubated with citrate buffer (pH 6.0) for 30 min (Vector Laboratories Inc., USA) in a microwave to improve antigen retrieval. After they were washed with Tris-buffered saline (TBS), the sections were incubated in normal goat serum for 30 min at room temperature to prevent nonspecific binding. Then, the sections from control, ALI/ARDS, and non-ALI/ARDS groups were incubated with a rabbit polyclonal primary antibody against SP-D (1:300 dilution; Ab203309, Abcam, UK) for 1 hr at room temperature. Next, the sections were incubated in a diluted biotinylated secondary antibody for 30 min followed by incubation for 30 min with VECTASTAIN ABC Reagent (Vector Laboratories Inc., USA). Visualization was achieved with diaminobenzidine (DAB) (Vector Laboratories Inc., USA) incubation for 5 min. The sections were counterstained with Mayer's hematoxylin and examined under a light microscope. The primary antibodies were omitted from the negative control sections, which were incubated with either TBS or normal goat serum. For semiquantitative analysis of SP-D-positive staining, each immunostained section was randomly assessed for staining intensity in 10 microscopic fields at a high magnification (400X). The SP-D staining intensity was scored as follows: 0 = negative staining, 1 = weak staining, 2 = moderate staining, and 3 = strong staining, as previously described [25]. Finally, the average staining intensity score for each experimental group was calculated and expressed as the mean ± standard error of the mean (SEM).

### 2.3. Enzyme-Linked Immunosorbent Assay (ELISA)

The levels of SP-D in the plasma and lung tissue were measured using a mouse SP-D ELISA kit according to the manufacturer's protocol (ab213890 Abcam, UK). Briefly, each plasma and protein sample was added to a 96-well plate and incubated for 90 min at 37°C. After discarding the contents in the wells without washing, 100 *μ*l of a biotinylated antibody was added to each well and incubated at 37°C for 60 min. After washing of the well plate with phosphate-buffered saline (PBS), avidin biotin peroxidase was added to each well and incubated for 30 min at 37°C. After a further washing step, tetramethylbenzidine (TMB) solution was added to the well plate as a substrate and incubated for 15-20 min at 37°C in the dark. Finally, the optical density in each well was measured at a wavelength of 450 nm in a microplate reader within 30 min after adding the stop solution. All the assays were performed in duplicate.

### 2.4. Western Blot Analysis

For Western blots, lung tissues were homogenized in cell lysis buffer (#9803, Cell Signaling Technology, USA) containing phenylmethylsulfonyl fluoride (Calbiochem, San Diego, CA), which is a protease inhibitor, to prepare the protein samples. The total protein concentration of the lung tissues was determined using a Bradford Protein Assay Kit (Bio-Rad Laboratories, USA) with bovine serum albumin (BSA) as the standard. Total protein (100 *μ*g) from each sample was resolved by reducing and separating by 12% SDS-polyacrylamide gel electrophoresis (SDS-PAGE) (Bio-Rad Laboratories, USA) at room temperature (RT). Then, the samples were transferred onto an activated polyvinylidene fluoride (PVDF) membrane (Bio-Rad Laboratories, USA). The membrane was blocked with 5% nonfat milk (Amresco, USA) in TBS-0.01% Tween 20 (TBS-T) for 2 hr at RT. The membrane was further incubated overnight at 4°C with a rabbit polyclonal antibody against SP-D (1:1000 dilution; Ab97849, Abcam, UK) as the primary antibody and a rabbit polyclonal antibody against *β*-actin (1:1000 dilution, Ab8227, Abcam, UK) as the positive control. After being washed with TBS-T, the membrane was incubated with HRP-conjugated goat anti-rabbit secondary antibodies (1:5000 dilution; ab6721, Abcam, UK) for 1 hr at RT. Finally, the protein bands on the membrane were detected using a chemiluminescent substrate (ECL Plus) kit (Amersham Biosciences, UK). The molecular weights of the detected protein bands were estimated using standard proteins (Bio-Rad Laboratories, USA) ranging from 10 to 250 kDa. Quantitative densitometric analysis of the gel bands was conducted using ImageJ software (version 1.6, NIH, Bethesda, MD), and the signal intensity of each band was normalized using the corresponding *β*-actin loading control. All the assays were performed in duplicate.

### 2.5. Statistical Analysis

The results are presented as the mean ± SEM. IBM SPSS scientific statistics version 23.0 software (SPSS, IL, USA) was used for statistical analyses. Data in this study was not normally distributed determined by the Kolmogorov–Smirnov test. Mann–Whitney* U* test was used to compare parasitemia among non-ALI/ARDS and ALI/ARDS groups. Comparisons between the control, non-ALI/ARDS, and ALI/ARDS groups were performed using the Kruskal–Wallis test. The correlations between the level of SP-D in the plasma and lung homogenates, and the level of SP-D in the plasma and the staining intensity score were determined by Spearman's rank correlation. The differences were considered statistically significant when* p* < 0.05.

## 3. Results

### 3.1. The Expression of SP-D in the Lung Tissues of Malaria-Infected Mice with ALI/ARDS

**Figure 1:**
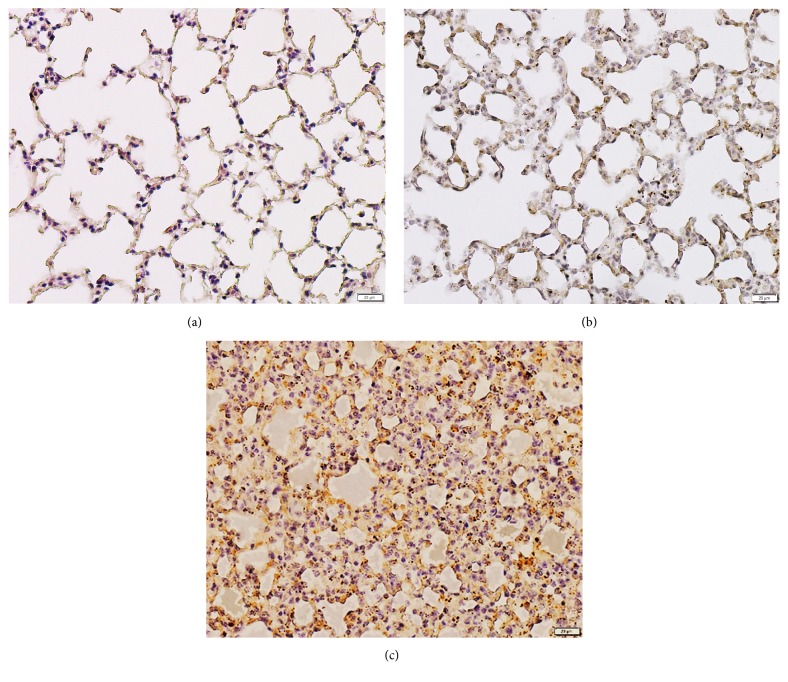
Representative photomicrographs of SP-D expression in the lung tissues. (a) Lung tissue of the mice in the control group, (b) lung tissue of the malaria-infected mice in the non-ALI/ARDS group, and (c) lung tissue of the malaria-infected mice with ALI/ARDS. All the images are magnified 400×. Scale bar = 20 *μ*m. Notice alveolar type I cells are thin and flat whereas alveolar type II cells are cuboidal. Alveolar macrophages are pleomorphic in shape, adhered to the internal surface of alveolar septum.

The results of the immunohistochemical studies demonstrated that the cytoplasmic positive staining for SP-D was located in the alveolar epithelial cells, especially alveolar type II cells and alveolar macrophages (Figure 1). The lung tissue of the malaria-infected mice with ALI/ARDS showed strong positive staining for SP-D (Figure 1(c)). In contrast, there were a few positively stained cells for SP-D in the lung tissues of the malaria-infected mice in the non-ALI/ARDS group (Figure 1(b)) and the mice in the control group (Figure 1(a)). For the quantification analysis, the mean staining intensity score for SP-D expression was significantly increased in the lung tissues of malaria-infected mice with ALI/ARDS (2.44 ± 0.05) compared with that in the malaria-infected mice in the non-ALI/ARDS group (1.24 ± 0.02) and the mice in the control group (1.22 ± 0.03) (all* p* < 0.05) (n = 5 mice per group).

**Figure 2:**
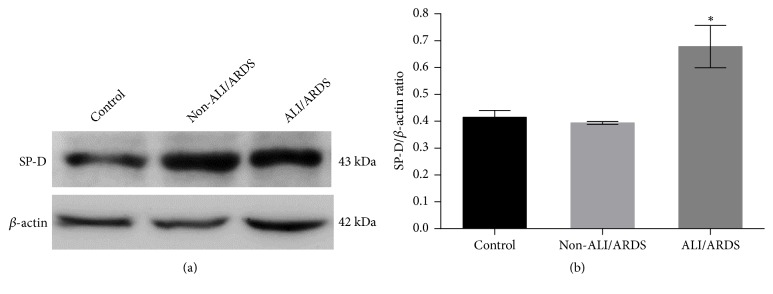
Western blot analysis of SP-D expression in the lung tissues from the malaria-infected mice with ALI/ARDS, malaria-infected mice in the non-ALI/ARDS group, and mice in the control group (n = 5 mice per group). (a) Representative immunoblot images for SP-D and (b) the quantification of SP-D expression by Western blot analysis. *∗*Significance of* p* < 0.05 compared among groups using Kruskal–Wallis test.

To confirm the expression of SP-D at the protein level, the expression level of SP-D in the lung tissues was detected by Western blot analysis with an SP-D antibody (Figure 2(a)). A band of SP-D (approximately 43 kDa) was detected in the lung tissues from all the groups, but the level of SP-D was increased in the lung tissue from mice in the ALI/ARDS group (Lane 3) compared to that in the lung tissue from the mice in the non-ALI/ARDS (Lane 2) and control groups (Lane 1). The quantification results indicated that the SP-D expression levels significantly increased in the lungs of the malaria-infected mice with ALI/ARDS compared with those in the lungs of the malaria-infected mice in the non-ALI/ARDS group and the mice in the control group (*p *< 0.05) (Figure 2(b)).

### 3.2. Increased Levels of SP-D in the Plasma and Lung Homogenates of the Malaria-Infected Mice with ALI/ARDS

**Figure 3:**
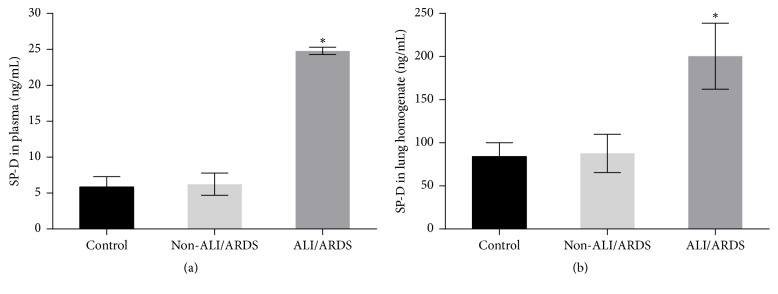
SP-D levels in the plasma and lung homogenate from the malaria-infected mice with ALI/ARDS, malaria-infected mice in the non-ALI/ARDS group, and mice in the control group (n = 5 mice per group). (a) SP-D in the plasma and (b) SP-D in the lung homogenates. *∗*Significance of* p* < 0.05 compared among groups using Kruskal–Wallis test. The data are presented as the mean ± SEM.

**Figure 4:**
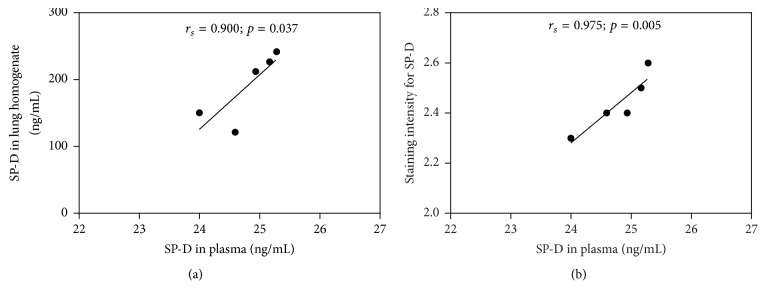
(a) Correlations between SP-D in the plasma and SP-D in the lung homogenate. (b) Correlations between SP-D in the plasma and the staining intensity scores for SP-D in the malaria-infected mice with ALI/ARDS (n = 5). The data were analyzed using Spearman's rank correlation test.

The levels of SP-D in the plasma and lung homogenates were measured by ELISA (Figure 3). The mean levels of SP-D in the plasma were significantly elevated in the malaria-infected mice with ALI/ARDS (24.79 ± 0.23 ng/mL) compared with those in the malaria-infected mice in the non-ALI/ARDS group (6.24 ± 0.69 ng/mL) and the mice in the control group (5.86 ± 0.64 ng/mL) (*p* = 0.009). In addition, the mean levels of SP-D in the lung homogenates were significantly elevated in the malaria-infected mice with ALI/ARDS (200.50 ± 17.15 ng/mL) compared with those in the malaria-infected mice in the non-ALI/ARDS group (87.71 ± 9.95 ng/mL) and the mice in the control group (84.07 ± 7.14 ng/mL) (*p* = 0.011). Spearman's rank correlation analysis revealed that there was a significant positive correlation between SP-D in the plasma and SP-D in the lung homogenate (*r*_s_ = 0.900,* p* = 0.037). Moreover, there was a significant positive correlation between the levels of SP-D in the plasma and the staining intensity score for SP-D (Spearman's rank correlation,* r*_s_ = 0.975,* p* = 0.005) (Figure 4).

## 4. Discussion

In our study, we used DBA/2 mice infected with* Plasmodium berghei* ANKA as an animal model of malaria associated with ALI/ARDS. The mice were classified as having ALI/ARDS based on the presence of pulmonary edema, alveolar hemorrhage, alveolar septal thickness, and hyaline membrane formation. These pathological findings corresponded to the histopathology of human ALI/ARDS reported by Punsawad et al. [25]. The mean parasitemia on the 13^th^ day after infection was 22.46% and 29.42% in the non-ALI/ARDS and ALI/ARDS groups, respectively (*p* = 0.008).

In the present study, we investigated the expression and circulation of the SP-D protein in a mouse model of malaria associated with ALI/ARDS. Our study demonstrated that positive immunoreactivity for SP-D was localized to the alveolar type II cells and alveolar macrophages. These findings are consistent with previous studies, which reported the location of SP-D positive staining in alveolar type II cells and alveolar macrophages [7, 20, 21]. The semiquantitative analysis showed that the mean staining intensity score for SP-D was significantly higher in the lung tissues of malaria-infected mice with ALI/ARDS than in the lung tissues of the malaria-infected mice in the non-ALI/ARDS group and the mice in the control group. Structurally, this protein consists of four structural domains, including a collagenous domain and a C-terminal carbohydrate recognition domain (CRD), which mediates interactions with pathogens, leading to cellular phagocytosis [15, 16]. During malaria infection, a previous study has revealed that proinflammatory cytokines induced by glycosylphosphatidylinositols (GPIs) of* Plasmodium falciparum* [26] and Hz [27] contribute to the pathogenesis of malaria. It is possible that the high expression level of SP-D may reflect the role of SP-D on the host defense mechanism via a CRD-dependent or CRD-independent mechanism, leading to the activation of anti-inflammatory pathways, enhanced phagocytosis in alveolar macrophages, and the activation of SP-D secretion in alveolar epithelial cells [15, 16]. Our findings support the important role of SP-D in the development of malaria-associated ALI/ARDS. However, the current study did not measure the time course of SP-D expression. A previous report on bleomycin-induced lung injury in a rat model demonstrated increased expression levels of SP-D in alveolar type II hyperplasia from days 3 to 10 that returned to the control level after day 10 [9]. High expression levels of SP-D may also enhance the regulation of inflammation and help with the clearance of malaria parasites. According to SP-D expression in alveolar macrophages, a previous study using ultrastructural analysis demonstrated that SP-D is present in the endocytic compartment in rat alveolar macrophages but not in biosynthetic organelles, indicating that SP-D is not produced by these inflammatory cells but rather SP-D is taken up by endocytosis [19, 20]. However, more* in vitro* and* in vivo* studies are needed to further understand the role of SP-D in alveolar macrophages during ALI/ARDS.

In the present study, an increased level of plasma SP-D was observed in the malaria-infected mice with ALI/ARDS compared with that in the malaria-infected mice in the non-ALI/ARDS group and the mice in the control groups. This finding is consistent with previous studies that showed increased serum SP-D levels in an experimental model of ALI [6, 7, 9, 10]. Previous reports demonstrated that the serum SP-D levels increased on days 3 to 28 after treatment with bleomycin [7, 9, 10], suggesting that serum SP-D could be useful for evaluating and monitoring signs of ALI. In the* Pseudomonas aeruginosa*-induced pneumonia and sepsis model in mice, compared to wild-type and humanized SP-D transgenic mice, mice lacking pulmonary SP-D are more susceptible to bacterial infection and exhibit more severe lung injury associated with bacterial pneumonia [28]. In accordance with the role of SP-D in inflammation, high levels of pulmonary interleukin-6 (IL-6) and tumor necrosis factor-*α* (TNF-*α*) and a 2-fold increase in the number of pulmonary monocytes/macrophages have been observed in* Sftpd*^−^/^−^ mice compared with those in wild-type mice [29]. In humans, previous studies have reported increased levels of plasma SP-D in patients with ALI/ARDS [5, 12, 14] that are associated with a higher mortality [12, 14]. In addition, the SP-D level has been shown to increase after the onset of ARDS to a maximum level on day 3, and the SP-D level remains elevated up to day 14, suggesting that SP-D can be used to classify patients with a high or low risk for progression to ARDS and/or death after the onset of ARDS [13]. In contrast, circulating SP-D levels are not associated with the presence of lung injury or the extent of lung contusions in patients with multiple injuries [30]. The current study confirmed the elevated expression level of SP-D in the lung homogenate of the malaria-infected mice with ALI/ARDS by Western blot analysis. However, the precise mechanism for the increased plasma SP-D levels after ALI/ARDS is unclear. The possible causes of plasma SP-D elevation are (i) an increase in augmented synthesis and/or impaired endocytosis of SP-D production [31], (ii) enhanced alveolar-capillary permeability in the lungs [12], and (iii) epithelial surfaces of several nonpulmonary organs that secrete SP-D as the potential sources of SP-D in the circulation [16, 21]. Further studies are required to clarify the mechanism for the alterations and regulation of SP-D levels in the circulatory system.

Some limitations of the present study should be addressed. We did not perform double staining with primary antibodies against the alveolar type II cells and lung macrophage markers. We did not determine the time courses of the BALF and plasma levels of SP-D in the malaria-infected mice with ALI/ARDS. An increased level of SP-D in BALF has been reported in a rat model of a bleomycin-induced lung injury [8, 9]. It has been reported that a biomarker panel could be used for precisely determining the diagnosis of ARDS [4, 5]. In addition, our study did not examine the performance of other biomarkers of lung epithelial injury and inflammation, such as club cell secretory protein (CC-16), receptor for advanced glycation end products (RAGE), IL-8, and IL-6.

## 5. Conclusions

The current study found increased expression levels of SP-D in lung tissues and high levels of plasma SP-D in malaria-infected mice with ALI/ARDS compared with those in the mice in the other groups. This study supports that elevated levels of plasma SP-D may be a useful biological confirmation of the presence of ALI/ARDS during malaria infection. Further investigations are needed for time course measurements of plasma SP-D and for the correlation of SP-D levels with clinical data in malaria patients with ALI/ARDS.

## Data Availability

The data used to support the findings of this study are included within the article.
